# A Child with X-Linked Agammaglobulinemia and Enthesitis-Related Arthritis

**DOI:** 10.1155/2011/175973

**Published:** 2011-06-01

**Authors:** Sukesh Sukumaran, Katherine Marzan, Bracha Shaham, Joseph A. Church

**Affiliations:** ^1^Division of Rheumatology, Children's Hospital Los Angeles, Los Angeles, CA 90027, USA; ^2^Division of Clinical Immunology and Allergy, Children's Hospital Los Angeles, Los Angeles, CA 90027, USA

## Abstract

X-linked agammaglobulinemia (XLA) is a primary immune deficiency characterized by recurrent bacterial infections and profoundly depressed serum immunoglobulin levels and circulating mature B cells. We describe a 12-year-old boy with XLA and enthesitis-related arthritis (ERA). To date, there has been a paucity of reports of noninfectious inflammatory arthritis in children with XLA. This case illustrates that functional B cells and/or immunoglobulin are not required for ERA pathogenesis. In addition, this case suggests a possible link between immune deficiency, immune dysregulation, and rheumatic illness.

## 1. Introduction

The adaptive immune response consists of humoral immunity mediated by B lymphocytes and cellular immunity maintained by T lymphocytes. Immunoglobulins, products of B-cell progeny, plasma cells are the main mediators of humoral immunity. B-cell deficiencies or dysfunction lead to recurrent bacterial infections with encapsulated organisms such as *S. pneumoniae, M. catarrhalis, and H. influenza* [1, 2]. Agammaglobulinemia was the first primary immunodeficiency to be described. In 1952, Colonel Ogden Bruton noted the absence of the “gammaglobulin” fraction on protein electrophoresis in a boy with recurrent bacterial sinopulmonary infections [3]. Subsequently, the gene responsible for XLA was identified and named Bruton's tyrosine kinase (*BTK*) at Xq21.3. This tyrosine kinase is required for maturation of the B-cell lineage. The prevalence of XLA has been estimated at 1 case per 250,000 individuals in the United States.

The recurrent bacterial infections typical of XLA begin after 6 months of age when the maternally acquired transplacental antibody levels decrease and the infant is unable to synthesize antibodies normally.

ERA is a rare autoimmune disease with an estimated prevalence of 0.1-0.2% in the general population. ERA usually presents with oligoarthritis or mild inflammatory back pain. Enthesitis, which refers to inflammation of the areas of attachment of the ligaments, tendons, and fascia to the bone, is an important feature of the disease.

## 2. Case Report

A 12-year-old Caucasian boy with XLA and a history of recurrent knee effusions presented to Rheumatology Service at nine years of age. He had been diagnosed with XLA at two years of age when he presented with recurrent methicillin-resistant *Staphylococcal aureus* (MRSA) skin abscesses, upper respiratory infections, and otitis media. He was found to have neutropenia (absolute neutrophil count 0) and very low serum immunoglobulin levels: IgG 36 mg/dL (normal 600–1200 mg/dL), IgA 7 mg/dL (normal 70–300 mg/dL), and IgM 15 mg/dL (normal 50 mg/dL–300 mg/dL). Circulating CD19+ lymphocytes were undetectable. Genetic testing demonstrated a previously described mutation in *BTK*.

Treatment with immunoglobulin replacement therapy resulted in rapid reversal of circulating neutrophil numbers. Maintenance therapy, currently with subcutaneous immunoglobulin infusions weekly, has prevented recurrence of severe infections.

The patient presented to the Rheumatology Service with a two-year history of recurrent left knee effusions. His first episode was at seven years of age when he presented with a one-month history of left knee pain and limp that was not associated with fever or trauma. He subsequently developed joint swelling and after one week presented to the Emergency Department where synovial fluid from the affected knee was negative for bacteria, fungus, ureaplasma, and mycoplasma (see Table 1). The synovial fluid was sent to well-equipped laboratory, and the fluid was sent by appropriate culture medium and was incubated for adequate period of time.

Eight months later, the patient's left knee swelling recurred two days after he had played on a trampoline. Synovial fluid cultures were again negative for an infectious etiology. Magnetic resonance imaging (MRI) was suggestive of a left distal femoral epiphyseal osteomyelitis. He was treated with clindamycin for six weeks and clinically improved. 

At nine and one-half and at 10 years of age the patient developed left knee swelling which was not associated with fever, redness, or limitation of activity. His symptoms resolved with oral tolmetin in the anti-inflammatory dose, and no further evaluations were performed for infectious etiologies.

At 12 years of age he presented with pain involving multiple joints. Four weeks prior, he had sustained trauma to his right foot which resulted in right big toe pain and swelling. Two weeks later, he developed pain in left hip and lower back and a week after, pain in his right shoulder. Ibuprofen failed to control or improve his symptoms, and he became unable to move his right shoulder or ambulate. Diagnostic studies for infections were again unhelpful. MRI of the right foot was consistent with posttraumatic joint effusion and marrow contusion, and pelvic MRI was suggestive of left hip inflammatory arthropathy and bilateral sacroiliitis. The patient improved on naproxen alone and was discharged home on hospital day three. 

He was evaluated at each episode to rule out infection and trauma. His ophthalmology examination was negative for ocular inflammation in multiple occasions.

Other than XLA, the patient had no other known medical conditions and no known bleeding disorders. He was up to date in immunizations up to 2 yrs of age and had no medication allergies. Family history was significant for colitis and spinal arthritis in a maternal aunt and psoriasis in a maternal cousin. The patient's half-brother also has XLA. There is no family history of bleeding disorders. Although he continued to receive immunoglobulin replacement for XLA, this did not prevent recurrent episodes of synovitis. The patient responded well to acute anti-inflammatory treatment, but he was nonadherent with maintenance therapy and followup outpatient visits. Other disease-modifying antirheumatic drugs (DMARDs) and biologic therapies that are indicated in the management of childhood ERA were not used because of his good response to tolmetin and the significant potential for increased risk for infections associated with his primary immune deficiency. The diagnosis of ERA was based on the patient's recurrent knee inflammation, lack of an infectious etiology, clinical enthesitis, sacroiliitis, HLA-B27 positivity, and a family history of psoriasis and colitis.

## 3. Discussion

ERA encompasses a heterogeneous group of inflammatory diseases characterized by axial and asymmetric peripheral arthritis, enthesitis, and at times, ocular, gastrointestinal, or cardiac manifestations. These disorders show a familial aggregation and are typically associated with HLA genes of the major histocompatibility complex (MHC), particularly HLA-B27. MHC class I molecules present antigenic epitopes to antigen-specific CD8+ cytotoxic T cells (CTLs) and thus determine which antigens will elicit a CTL response.

In the absence of a functional Bruton's tyrosine kinase gene, mature B cells that express surface Ig and CD19 are few to absent. The absence of CD19+ cells is readily detected with fluorocytometric assays, and this finding strongly suggests XLA in an affected male. *BTK* mutation analysis confirms the diagnosis.

This case illustrates that mature B-cells and immunoglobulins are not required for ERA pathogenesis. In addition, this case provides a possible link between immune deficiency, immune dysregulation, and rheumatic illnesses. To our knowledge ours is the first report of ERA and XLA. 

Anti-B cell therapies, such as Rituximab have recently been shown to be an effective therapy in selective forms of noninfectious inflammatory arthritis [4]. However in this child, inflammatory arthritis developed in the absence of B-cell function. The exact mechanism by which this occurred is not known but it is likely that T-cell dysregulation, either due to or independent of the absence of B cells, was primarily responsible for ERA in our patient.

##  Conflict of Interests

The authors declare no conflict of interests.

## Figures and Tables

**Table 1:** Laboratory evaluation of the synovial fluid and blood performed in a 12-year-old boy with X-linked agammaglobulinemia and recurrent knee effussions over a period of 5 years.

Lab investigations	04/2010	03/2008	08/2007	03/2006	07/2005
Synovial fluid WBC (cells/mm^3^)	110	30,960	27,000	40,000	220
Synovial fluid culture	Neg	Neg	Neg	Neg	Neg
Crypto/Cocci Ag/fungal stain and Cx	Neg	Neg	Neg	Neg	Neg
Atypical mycobacterium/mycoplasmas/ureaplasma	Neg	Neg	Neg	Neg	Neg
AFB	Neg	Neg	neg	neg	neg
ESR mm/hr-CRP mg/dL	60/5.1	41/4.1	30/13.5	34/7.5	40/2.3
Blood culture and viral cultures	Neg	Neg	Neg	Neg	Neg
